# 2377. The Influence of the COVID-19 Infodemic on Vaccine Hesitancy: A Prospective Survey-Based Study

**DOI:** 10.1093/ofid/ofad500.1998

**Published:** 2023-11-27

**Authors:** Cameron Quon, Macey Walker, Lisa Graves

**Affiliations:** Western Michigan University Homer Stryker M.D. School of Medicine, Santa Clarita, California; Western Michigan University Homer Stryker M.D. School of Medicine, Santa Clarita, California; Western Michigan University Homer Stryker M.D. School of Medicine, Santa Clarita, California

## Abstract

**Background:**

The COVID-19 pandemic was an "infodemic" of accurate and inaccurate information as termed by the World Health Organization. Media engagement has been correlated with greater belief in misinformation and lower vaccination rates.

The aim of this study was to investigate how individuals make decisions regarding the COVID-19 vaccine with a particular focus on specific media outlet consumption.

**Methods:**

We conducted an online survey via the Western Michigan Homer Stryker M.D. School of Medicine Facebook account from August 1, 2021 to August 30, 2021. The primary outcome was the choice to take the COVID-19 vaccine. Respondents were assigned a media score based on the companies they used to receive COVID-19 information. The score was calculated based on a Pew Research Center study on the political leaning of various news outlets.

A Sampling of the Political Spectrum of Media Outlets

**Figure:**
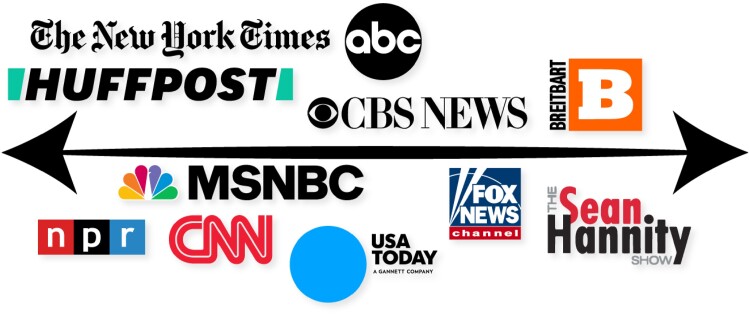

Some of the major media outlets assessed in our survey with the left being more liberal and right being more conservative.

**Results:**

There were 1,757 responses with a prevalence of 89.6% vaccine acceptors. Respondents were predominantly White, female, and liberal with an average age of 44. There was a 1.06 (95% CI 1.04, 1.07) multiplicative increase in odds of choosing the vaccine for every point towards more liberal media consumption. Both media score (c=0.77) and political leaning scale (c=0.81) had a strongly positive correlation with vaccination choice. Attitudes of vaccine acceptors and non-acceptors aligned with the viewpoints of their friends and family. While both groups had good relationships with their doctors, there were no significant differences in vaccine choice.

Respondent Demographics and Political Spectrum

**Figure:**
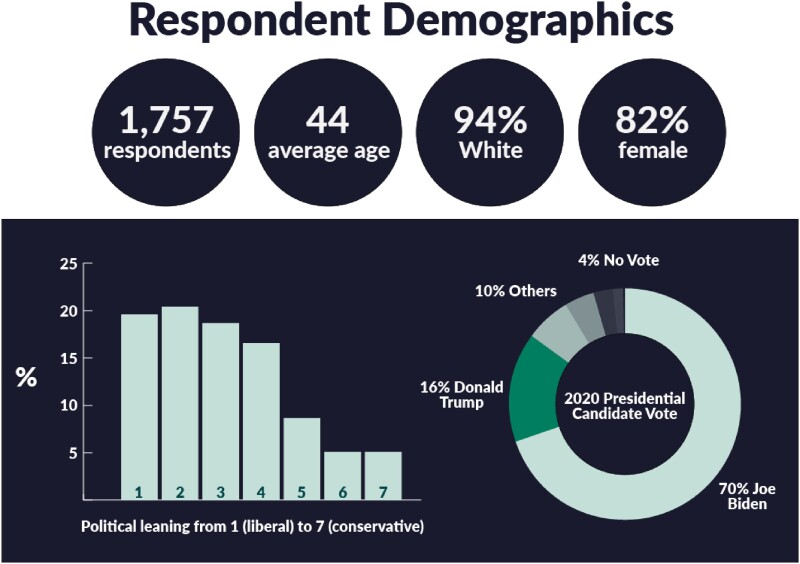

The demographics and political spectrum of the survey's respondents.

Likelihood of choosing the vaccine

**Figure:**
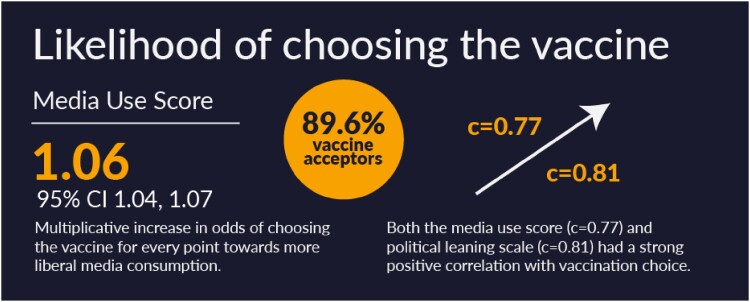

The prevalence of vaccine acceptors, the odds ratio for media use, and the correlation between media and vaccination and political affiliation and vaccination.

**Conclusion:**

This study demonstrates that individuals who prefer more liberal leaning media outlets are more likely to choose to receive the vaccine. They also suggest the physician's voice must compete with the media in terms of vaccine messaging.

While this study is limited by a respondent demographic that did not necessarily reflect that of the country, it is at least reflective of those who engage with the medical school's outreach. Performing a study of this nature within local communities can provide population insights enabling customized communication strategies.

Future studies should explore ways physicians can use media to enhance their voice and counter misinformation. Access to reliable information about the COVID-19 vaccine is critical in helping individuals make informed decisions about vaccination.

**Disclosures:**

**All Authors**: No reported disclosures

